# Structure–Function Relationships in the Rodent Streptozotocin-Induced Model for Diabetic Retinopathy: A Systematic Review

**DOI:** 10.1089/jop.2021.0128

**Published:** 2022-05-03

**Authors:** Inesa Lelyte, Zubair Ahmed, Simon Kaja, Giedrius Kalesnykas

**Affiliations:** ^1^ Research and Development Division, Experimentica Ltd., Kuopio, Finland.; ^2^ Institute of Inflammation and Ageing, and University of Birmingham, Birmingham, United Kingdom.; ^3^ Center for Trauma Sciences Research, University of Birmingham, Birmingham, United Kingdom.; ^4^ Department of Ophthalmology and Molecular Pharmacology and Neuroscience, Stritch School of Medicine, Loyola University Chicago, Maywood, Illinois, USA.; ^5^ Experimentica Ltd., Research and Development Division, Forest Park, Illinois, USA.; ^6^ Experimentica Ltd., Research and Development Division, Vilnius, Lithuania.

**Keywords:** streptozotocin, diabetic retinopathy, glucose level, electroretinogram, optical coherence tomography, fluorescein angiography

## Abstract

The streptozotocin (STZ)-induced rodent model is one of the most commonly employed models in preclinical drug discovery for diabetic retinopathy (DR). However, standardization and validation of experimental readouts are largely lacking. The aim of this systematic review was to identify and compare the most useful readouts of STZ-induced DR and provide recommendations for future study design based on our findings. We performed a systematic search using 2 major databases, PubMed and EMBASE. Only articles describing STZ-induced DR describing both functional and structural readouts were selected. We also assessed the risk of bias and analyzed qualitative data in the selected studies. We identified 21 studies that met our inclusion/exclusion criteria, using either rats or mice and study periods of 2 to 24 weeks. Glucose level thresholds used to define hyperglycemia were inconsistent between studies, however, most studies used either 250 or 300.6 mg/dL as a defining criterion for hyperglycemia. All included studies performed electroretinography (ERG) and reported a reduction in a-, b-, or c-wave and/or oscillatory potential amplitudes. Spectral-domain optical coherence tomography and fluorescein angiography, as well as immunohistochemical and histopathological analyses showed reductions in retinal thickness, vascular changes, and presence of inflammation. Risk of bias assessment showed that all studies had a high risk of bias due to lack of reporting or correctly following procedures. Our systematic review highlights that ERG represents the most consistent functional readout in the STZ model. However, due to the high risk of bias, caution must be used when interpreting these studies.

## Introduction

Diabetic retinopathy (DR) occurs in more than one-third of individuals with diabetes and is one of the leading causes of blindness in individuals 24 to 74 years of age.^1^ With increasing human life expectancy and rising numbers of diabetics, it is estimated that the number of people with DR worldwide will grow from 126.6 million diagnosed in 2010 to 191.0 million cases by 2030.^2^ Almost all patients who are diagnosed with type I diabetes will develop some degree of DR within 2 decades after diagnosis; for type II diabetes this probability increases up to 80% at 20 years after disease onset.^3^ Hyperglycemia, oxidative stress, anemia, pericyte loss, apoptosis of endothelial cells, thickening of the basement membrane, and other mechanisms are considered to play an important role in the pathogenesis of DR.^4^ If DR is left untreated, it results in diabetic macular edema (DME) and proliferative DR (PDR), which leads to total loss of vision.^1^

Thus, there is an urgent need for novel therapeutics that can slow down the progression of disease and prevent the development of end-stage DR, especially DME and PDR.

Animal models that mimic the pathophysiology of DR have contributed significantly to the understanding of the etiology and progression of this condition, and validated and standardized models are required for drug discovery and development. Historically, DR animal model studies have relied primarily on histological and immunohistochemical readouts.^5,6^

In addition, various molecular and biochemical techniques, including quantitative polymerase chain reaction (qPCR), western blot, TUNEL assay, as well as protein, enzyme, and cytokine assays are carried out to study the development of DR in diabetic animals.^7–9^ Research shows that fluorescein angiography (FA), spectral-domain optical coherence tomography (SD-OCT), and electroretinography (ERG) in animal models of DR could potentially help to detect early changes of DR, and could aid in developing novel, more potent drugs for an early treatment of DR.^10–15^

The most commonly used rodent model in drug discovery utilizes streptozotocin (STZ) administration to induce chronic hyperglycemia and DR by causing pancreatic β cell loss.^16^ DR phenotypes observed in STZ-induced rodents include thinning of retinal layers,^17,18^ loss of retinal cells,^19^ neovascularization,^17^ and increased inflammation.^20^ Historically, preclinical studies relied on morphological changes as detected using *post mortem* tissue processing, histological and immunohistochemical staining, and morphometric analyses.

With advancement in functional assessments and, in particular, adaptation of clinically used *in vivo* imaging modalities, detection of early phenotypes reminiscent of subclinical and/or non-PDR is now possible in rodents. However, both functional and imaging modalities rely on a clear optical path and measurements can be confounded or even become impossible as animals develop diabetic cataracts. Therefore, accurate and early detection of DR-related changes by noninvasive techniques requires strict validation to identify the optimal balance between disease progression, accuracy of assessment of structure–function relationships and magnitude of the therapeutic window for drug discovery.

These considerations are of particular importance as early phenotypes in the STZ model that can be studied using in-life imaging modalities and electrophysiological recordings identify and assess mostly subclinical pathological changes. Furthermore, while OCT and FA, and more recently, optical coherence tomography angiography (OCT-A) are routinely used to diagnose the various disease stages of DR, electrophysiology is not typically used in ophthalmic practice.

Despite rapid development of noninvasive methods and their applications, the STZ-induced DR model lacks standardized experimental paradigms and readouts. Therefore, the goal of this study is to perform systematic literature review of the literature in search of standardized readouts or experimental paradigms, which would be useful in future STZ studies.

## Methods

### Literature search

We followed a comprehensive search strategy to obtain published articles by using the recommendations for the Preferred Reporting Items for Systematic Reviews and Meta-Analyses (PRISMA) reporting guidelines.^21^ Two major databases, PubMed and EMBASE, were investigated to identify articles suitable to STZ-induced DR animal model, from inception to 14th of August 2020. The searched items were used in combination: STZ OR streptozotocin AND diabetic retinopathy OR FA OR fluorescence angiography OR OCT OR optical coherence tomography OR ERG OR electroretinogram OR VFP OR vitreofluorophotometry. Search terms were queried in both databases.

### Inclusion and exclusion criteria

Records were included in this systematic review if they met the following inclusion criteria: (1) studies in English, (2) published in the last 10 years (starting 2010), (3) articles in peer-reviewed scientific journals, and (4) studies conducted on rodents only. We excluded the following: (1) Meta-analysis, review and systematic review articles, (2) clinical and *in vitro* studies, and (3) studies in languages other than English.

### Data collection

Two reviewers (I.L. and Z.A.) independently conducted the literature search based on the titles and abstracts. Any discrepancies were resolved by discussion and if needed, consultation with a third reviewer (S.K). Relevant articles had to meet the above inclusion/exclusion criteria. Records were imported into Excel, duplicates removed, titles and abstracts searched manually, and full text of potential studies was retrieved.

### Data extraction and synthesis

The following data are extracted: (1) study characteristics; (2) induction of DR; and (3) outcome measures in studies.

### Risk of bias assessment

To assess bias in the included studies, we used the SYRCLE risk of bias tool for animal studies, which is based on the Cochrane Collaboration's tool for assessing risk bias in randomized controlled trials.^22,23^ Bias assessments were carried out by 2 independent reviewers (I.L. and Z.A) and any discrepancies were resolved after discussion. Risk of bias was assessed using 10 criteria in the SYRCLE risk of bias tool that is related to 6 types of bias: selection bias, performance bias, detection bias, attrition bias, reporting bias, and other sources of bias.^22^

### Statistical analysis

Descriptive statistics were used to characterize included studies. The kappa statistic was used to assess interrater agreement between the 2 reviewers. Since included studies did not contain sufficient quantitative data, a meta-analysis was not possible and hence a qualitative comparison between studies was performed.

## Results

### Study selection

**FIG. 1.**
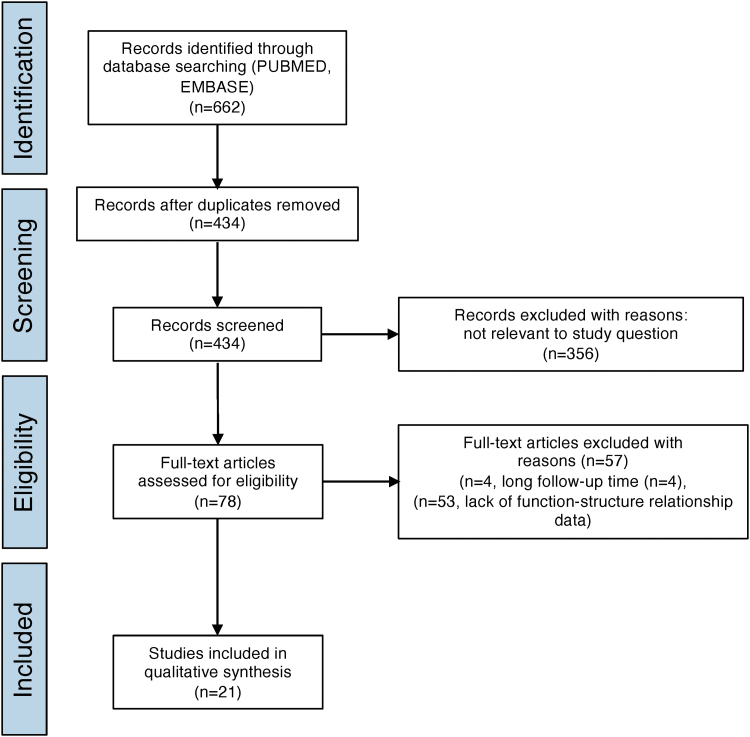
Prisma flow chart illustrating the process of literature search.

A systematic search according to our strategy identified a total of 622 studies obtained from PubMed and EMBASE (Fig. 1). After removing duplicates, 434 studies were screened by title and abstract and 356 were excluded. Seventy-eight articles were considered acceptable, of which 51 studies were excluded after full-text screening due to either a long follow-up time (*n* = 4) or lack of structure–function relationship readouts (*n* = 51). Ultimately, 21 studies were included in the qualitative synthesis. Section may be divided by subheadings. It should provide a concise and precise description of the experimental results, their interpretation, as well as the experimental conclusions that can be drawn.

### Study characteristics

From the included studies, 11 were conducted in rats (8 in Sprague-Dawley rats, 2 in Wistar rats, 1 study in Brown Norway rats), whereas the remaining studies were conducted in mice (3 in C57BL6, 4 in C57BL6/J; 2 in BALB/c, 1 in B6.129S4) (Table 1). Although all studies used STZ to induced DR, each one considered different glucose levels as diabetic. In addition, follow-up times also varied from 2 to 24 weeks post-DR induction.

**Table 1.** Characteristics of the Included Studies

Study	Species (animal number)	STZ injection	Follow-up time in weeks	Glucose level considered as diabetic (mg/dL)	Functional readouts	In vivo imaging	Postmortem analyses
He et al.^36,a^	Sprague-Dawley rats (*n* = 30)	Single intraperitoneal injection of STZ (60 mg/kg)+high-fat and high-calorie diet	4	300.6	Full-field ERG	OCT	Antioxidant parameters: the concentration of antioxidant SOD Inflammatory parameters: levels of proinflammatory cytokines (TNF-α and IL-6) Immunofluorescence and western blot assay: expression VEGF-α, ALDH2, and expression of Nrf2 and Sirt1.
Qiu et al.^43^	Brown Norway rats (*n* = 48)	Single intraperitoneal injection of STZ (50 mg/kg)	8	>350	Flash ERG	OCT	Evans Blue extravasation: retinal vascular permeability Leukostasis assay: number of adherent leukocytes Western blot analysis: levels of VEGF, ICAM-1, and β-actin;
Sergeys et al.^31^	C57BL/6J mice (*n* varies from 4 to 18 depending on the readout)	5 sequential daily intraperitoneal injections of STZ (50 mg/kg)	24	>250	Full-field ERG	FA, OCT	Leukostasis assay: number of adherent leukocytes H&E staining: observation of general retinal and the optic nerve head morphology Neurodegeneration: number of RGC (RBPMS) and cholinergic amacrine cell (ChAT); Inflammation and Gliosis: *n*umber of macrophages (F4/80); and Muller cell (vimentin)
Liao et al.^30,b^	C57BL/6 mice (*n* = 25)	5 sequential daily intraperitoneal injections of STZ (55 mg/kg) + whole-body hypoxia (10% O_2_)	4	>250	Flash ERG	O CT, FA, FP	H&E staining: *m*orphology of the retina TUNEL assay: *m*orphology of the retina. Number of INL and ONL cells Western blot analysis: expression of inflammatory and junction proteins (BAX, COX-2, iNOS) MTT assay: viability of RPE cells
Chang et al.^28^	C57BL/6J mice (*n* = 37)	3 sequential daily intraperitoneal injections of STZ (100 mg/kg)	12	>250	Flash ERG	FA	Immunofluorescent staining: observation of mitochondrial processes with DRP1, MFN2, and MCU
Feng et al.^29^	Sprague-Dawley rats (*n* = 90)	Single intraperitoneal injection of STZ (60 mg/kg)	4	>250	Full-field ERG	No data	BRB breakdown: extravasation of albumin; H&E staining: thicknesses of the IPL, INL and ONL, number of RGC; ELISA: activities of antioxidant enzyme (SOD, GSHPx, GSSGRed, and GSHTrans); and examination of oxidative stress (levels of 4-HNE and 8-OhdG); Western blot analysis: levels of BDNF and synaptophysin
Tarchick et al.^33,c^	Mice with inactivated insulin receptor (IR) B6.129S4 (FVB)-Insrtm1Khn/J (n varies from 9 to 18 depending on the readout)	3 sequential daily intraperitoneal injections of STZ (55 mg/kg)	4	>250	Flash ERG	No data	Light micrographs: the distance from OLM to ILM, the length of OS, IS, and ONL, and thickness of RPE was measured. DHE staining: measurement of reactive oxygen species
Nagai et al.^24^	Wistar rats (*n* = 7)	2 sequential daily intraperitoneal injections of STZ (100 mg/kg)	6	267.6	Flash ERG	No data	ELISA: level of VEGF; H&E staining: the distance between RGC to the outer granule layer
Deguchi et al.^25^	Wistar rats (*n* = 6)	2 sequential daily intraperitoneal injections of STZ (100 mg/kg)	2	No data	Flash ERG	No data	H&E staining: distance between the GCL and the distal border of the ONL
Hernández et al.^26^	Sprague-Dawley rats (*n* = 24)	Single injection of STZ (60 mg/kg) into the tail vein	2	>250	Flash ERG	No data	Assessment of glial activation: expression of GFAP; TUNEL assay: assessment of apoptosis (number of retinal apoptotic cells); Western blot analysis: observation of proapoptotic and survival signaling; Real-time PCR and immunohistochemistry: expression of GLAST.
Wang et al.^41^	C57BL/6 mice (*n* = 36)	3 sequential daily intraperitoneal injections of STZ (75 mg/kg)	5	300.6	Focal ERG	FA	Leukostasis: number of adherent leukocytes and vascular endothelial cells; Western blot analysis: Levels of GFAP, VEGF and ICAM-1 levels; BRB breakdown: extravasation of albumin; DHE assay: measurement of MDA, SOD, and ROS formation
Tarchick et al.^33^	C57BL/6J mice (*n* ≥ 10)	3 sequential daily intraperitoneal injections of STZ (30 mg/kg)	4	≥250	dc-ERG	No data	Light microscopy: length of the inner segment, outer segment, and ONL; thickness of RPE; PNA staining: Number of rod and cone photoreceptor nuclei per column
Lv et al.^38^	BALB/c Mice (*n* = 25)	5 sequential daily intraperitoneal injections of STZ (50 mg/kg)	2	≥300.6	Full-field ERG	No data	H&E staining: The thickness of GCL, INL, ONL, and total retina
Zhang et al.^34^	Sprague-Dawley rats (*n* = 24)	Single STZ injection of STZ (30 mg/kg)	12	>199.8	Flash ERG	No data	H&E staining: thickness of total retina, thickness of INL and ONL layers, and cell number in the INL and ONL; TUNEL assay: apoptosis assessment; Immunohistochemistry: expression of Bcl-2, Bax.
He et al.^37^	Sprague-Dawley rats (*n* = 40)	Single intraperitoneal injection of STZ (35 mg/kg)	10.2	>300.6	Full-field ERG	OCT	Western blot analysis: expression of VEGF, GFAP, and VCAM-1; Levels of GLU, TC, and TG; Levels of GSH-Px, SOD, and CAT.
Liao et al.^32^	C57/BL6 mice (*n* = 40)	5 sequential daily intraperitoneal injections of STZ (55 mg/kg)	5	No data	Flash ERG	FA + FP	Trypsin-digested retinal whole mounts: number of acellular capillaries H&E staining: number of preretinal neovascular cells; Thicknesses of the IPL, INL, OPL, and ONL; RT-qPCR and western blot analysis: expressions of VEGF; levels of proinflammatory cytokines, including TNF-a, IL-1b, IL-6, IL-18, and IL-12;
Goharinia et al.^35^	Sprague-Dawley rats (*n* = 8)	Single intraperitoneal injection of STZ (60 mg/kg)	8	300	Flash ERG	No data	H&E staining: number of RGCs
Ren et al.^40^	BALB/c mice (*n* = 20)	Single intraperitoneal injection of STZ (80 mg/kg)	4	≥300.6	Full-field ERG	No data	H&E staining: the thickness of the whole retina, ONL, INL, and RGC layer; Trx expression.
Piano et al.^39^	C57BL/6J mice (*n* = 20)	Single intraperitoneal injection of STZ (150 mg/kg)	12	>300.6	Flash ERG	No data	Cryosections: total retinal thickness; Vascular area: deep plexus vessels; neovascularization; Western blot analysis: rhodopsin and cone-opsin content; TUNEL staining: apoptosis of rod photoreceptors; Western blot analysis: level of Caspase-3 and GAPDH
Yee et al.^27,d^	Sprague-Dawley rats (*n* = 56)	Single injection of STZ (53 mg/kg) into the tail vein+specific fish oil or safflower oil diet	20	270	Flash ERG	No data	Toluidine Blue staining: thickness of ONL, INL, IPL layers, and total retina. Retinal fatty acid analysis: identification of fatty acids in retina Immunolabeling: level of GFAP
Wang et al.^42^	Sprague-Dawley rats (*n* = 16)	Single intraperitoneal injection of STZ (35 mg/kg) + high-sucrose/high-fat diet	17	300.6	fVEP	OCT	H&E staining: number of RGC; Expressions of GFAP and number of activated astrocytes; PAS assessment: retinal microvascular changes, ghost pericytes.

Reasons for study exclusions.

^a^ No control group (only a group with STZ).

^b^ STZ administration was combined with whole-body hypoxia (10% O_2_) for quicker diabetes induction.

^c^ Mice with inactivated insulin receptor.

^d^ STZ administration with specific oil diet.

BAX, Bcl-2-associated X protein; BRB, blood–retinal barrier; ChAT, choline acetyltransferase; COX-2, cyclooxygenase-2; DHE, dihydroethidium; DR, diabetic retinopathy; ELISA, enzyme-linked immunosorbent assays; ERG, electroretinography; FA, fluorescein angiography; fVEP, functional visually evoked potentials; GAPDH, glyceraldehyde 3-phosphate dehydrogenase; GCL, ganglion cell layer; GFAP, glial fibrillary acidic protein; H&E, Hematoxylin and Eosin; ICAM-1, intercellular adhesion molecule-1; IL, interleukin; INL, inner nuclear layer; iNOS, inducible nitric oxide synthases; IPL, inner plexiform layer; MCU, mitochondrial calcium uniporter; OCT, optical coherence tomography; ONL, outer nuclear layer; OPL outer plexiform layer; PAS, Periodic acid–Schiff; PCR, polymerase chain reaction; RGC, retinal ganglion cell; RT-qPCR, reverse transcription quantitative polymerase chain reaction; SOD, superoxide dismutase; STZ, streptozotocin; TNF-α, tumor necrosis factor-a; VCAM-1, vascular cell adhesion molecule-1; VEGF, vascular endothelial growth factor.

### STZ injection

The majority of included studies with rats used single STZ injection to induce DR. Only Nagai et al.^24^ and Deguchi et al.^25^ used 2 STZ injections on 2 consecutive days. However, the volume of administered STZ for rats varied from 30 to 100 mg/kg. As for the type of injections, almost in all studies, researchers performed intraperitoneal injections, except Hernández et al.^26^ and Yee et al.,^27^ who administered STZ through the rat's tail vein. In contrast, the number of STZ injections in mice studies were more variable. From collected studies, 4 of them had 5 STZ injections, 3 studies had 3 injections, and 2 single STZ injections. The volume of STZ injections for mice varied from 50 to 150 mg/kg. All STZ injections to mice were performed intraperitoneally.

### Glucose level considered as diabetic

Out of all included articles, 8 studies considered rodents as diabetic, which had equal or greater than 250 mg/dL of glucose.^26,28–33^ While Zhang et al.^34^ considered a threshold of 199.8 mg/dL (the equivalent of 11.1 mmol/L) of glucose as diabetic for Sprague-Dawley rats. However, for Nagai et al.^24^ and Yee et al.^27^ glucose level had to rise more than 250 mg/dL to consider rats as diabetic, 267.6 and 270 mg/dL (the equivalent of 15 mmol/L), respectively. Other 7 research groups considered rodents with induced diabetes with equal or greater than 300.6 mg/dL (the equivalent of 16.7 mmol/L) of glucose.^35–42^ While for Qiu et al.^43^ glucose level in animals had to hit 350 mg/dL, to be considered as diabetic. Only Deguchi et al.^25^ and Liao et al.^30^ presented no data of glucose level in rodents.

### Functional readouts

All studies used either flash electroretinography or full-field ERG, direct-coupled electroretinography (DC-ERG) or visual evoked potentials (VEP).

### *In vivo* imaging

From included articles, six^30,31,36,37,42,43^ performed OCT to observe structural changes of the retinas and to evaluate total retinal thickness and the thickness of individual retinal layers. The other 5 studies^28,30,31,39,43^ used FA to monitor morphological and pathological changes in the eye fundus. Liao et al.^30^ and Liao et al.^32^ performed fundus photography (FP) in addition to FA. The rest of included articles did not use any *in vivo* imaging.

### Postmortem analysis

The included studies used a number of different postmortem analyses, including Hematoxylin and Eosin (H&E) and Toluidine Blue staining to quantify retinal thickness, western blot assays, and enzyme-linked immunosorbent assay to detect specific proteins in retina; qPCR to evaluate gene expression, leukostasis assays to count the number of adherent leukocytes, Evans Blue extravasation to quantify blood–retinal barrier breakdown, and to measure retinal vascular permeability, trypsin-digested retinal whole mounts for quantification of acellular capillaries and pericyte loss, as well as TUNEL and MTT assays to assess viability of retina cells.

### Subgroup analysis

**FIG. 2.**
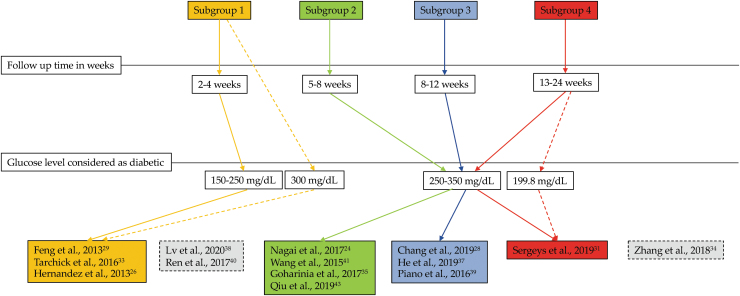
Overview of subgroup analysis. The majority of the studies fell into subgroups 1–4 based on follow-up time (*solid arrows*). However, some studies fell into specific subgroups, although the authors of those studies considered different levels of blood glucose as diabetic (*dotted arrows*).

We grouped all of the selected studies into 4 subgroups based on the follow-up time after STZ administration: subgroup 1 had a follow-up time of 2–4 weeks, subgroup 2 had a follow-up time of 5–8 weeks, subgroup 3 had a follow-up time of 8–12 weeks, and subgroup 4 had a follow-up time of 13–24 weeks (Fig. 2). All studies in subgroup 1 considered 150–250 mg/dL glucose levels as diabetic, while studies in subgroups 2–4 considered glucose levels of 250–350 mg/dL as diabetic. Although some studies fell into subgroup 1 with respect to follow-up time, they considered 300 mg/dL of glucose levels as diabetic. In one study^25^ no data for glucose levels were reported. Insulin was administered only in the study by Tarchick et al.^44^ every other day. The study by Liao et al.^32^ fell into subgroup 2 but did not report glucose levels they considered to be diabetic. The study by Zhang et al.^34^ fell into subgroup 3 because of follow-up time, but they considered glucose levels of 198.6 mg/dL as diabetic.

### Outcomes of selected studies

#### Glucose levels and body weights

All studies reported an increase in glucose levels in STZ-induced animals^24–26,28,29,31–35,37–43^ (Table 2). However, only 4 studies provided raw numbers of glucose levels. The increase in glucose levels in those studies was species dependent. In Sprague-Dawley rats, the glucose level increased by fourfold after a single STZ administration,^29,35^ whereas a threefold increase was found in C57BL/6J mice after single STZ administration,^39^ and after 5 sequential daily intraperitoneal injections of STZ^31^ (Table 2).

**Table 2.** Outcomes of Studies: Glucose Levels and Body Weights

Study	Species	Glucose level (mg/dL)	Body weight (g)
Chang et al.^28^	C57BL/6J mice	Increased (no raw data)	Increased (no raw data)
Deguchi et al.^25^	Wistar rats	Increased (no raw data)	Decreased (no raw data)
Feng et al.^29^	Sprague-Dawley rats	Increased (Healthy control rat 93.6 ± 23.4, and STZ induced 390.6 ± 55.8)	Decreased (Healthy control rat 289.9 ± 5.2 and STZ induced 171.9 ± 14.5)
Goharinia et al.^35^	Sprague-Dawley rats	Increased (Healthy control rat 112 ± 4 and STZ induced 443 ± 24)	Decreased (Healthy control rat 268 ± 9 and STZ induced 178 ± 7)
He et al.^37^	Sprague-Dawley rats	Increased (no raw data)	N/A
Hernández et al.^26^	Sprague-Dawley rats	Increased (no raw data)	N/A
Liao et al.^32^	C57/BL6 mice	Increased (no raw data)	Decreased (no raw data)
Lv et al.^38^	BALB/c Mice	Increased (no raw data)	N/A
Nagai et al.^24^	Wistar rats	Increased (no raw data)	Decreased (no raw data)
Piano et al.^39^	C57BL/6J mice	Increased (Healthy control mice 135 ± 7.2 and STZ induced 502.2 ± 27)	Decreased (no raw data)
Qiu et al.^43^	Brown Norway rats	Increased (no raw data)	N/A
Ren et al.^40^	BALB/c mice	Increased (no raw data)	N/A
Sergeys et al.^31^	C57BL/6J mice	Increased (Healthy control mice 180 ± 8 and STZ induced 477 ± 29)	Decreased (Healthy control mice 30.8 ± 0.7 and STZ induced 22.7 ± 0.5)
Tarchick et al.^33^	C57BL/6 mice	Increased (no raw data)	Decreased (no raw data)
Wang et al.^41^	C57BL/6 mice	Increased (no raw data)	Increased (no raw data)
Wang et al. ^42^	Sprague-Dawley rats	Increased (no raw data)	N/A
Zhang et al.^34^	Sprague-Dawley rats	Increased (no raw data)	Decreased (no raw data)

N/A, data not available.

Body weight of STZ-induced animals decreased in the majority of studies^24,25,29,31,32,34,35,39,41,44^ (Table 2). However, in 2 studies, the authors found body weight gains in diabetic C57BL/6J mice as compared with healthy control groups.^28,41^ Similarly, as with blood glucose level data, one mouse study^31^ and 2 rat studies^29,35^ reported raw body weight data showing almost a twofold reduction in body weight after STZ injections, whereas others did not reveal any raw data,^24,25,28,32–34,39,41^ or did not record body weight changes at all^26,37,38,40,42,43^ (Table 2).

#### Visual function: ERG and VEP

All studies showed deterioration of visual function in STZ animals as compared with healthy controls either in amplitude reduction in a-, b-, c-wave and/or oscillatory potential (OP) as assessed by ERG, or in decrease of N and P wave amplitudes in VEP measurements. However, only 2 studies provided numerical values of a-wave, b-wave, and OP amplitudes expressed as percentage decrease in STZ-injected animals in comparison with healthy control animals (50% for a-wave, 70%–80% for b-wave, and 50%–60% for OP amplitudes)^37,38^ (Table 3).

**Table 3.** Outcomes of Studies: Electroretinography and Visually Evoked Potential Measurements

Sudies	Species	a-wave amplitude	b-wave amplitude	c-wave amplitude	OPs amplitude	VEP: amplitude of N and P waves
Chang et al.^28^	C57BL/6J mice	Decreased (no raw data)	Decreased (no raw data)			
Deguchi et al.^25^	Wistar rats	Decreased (no raw data)	Decreased (no raw data)		Decreased (no raw data)	
Feng et al.^29^	Sprague-Dawley rats		Decreased (no raw data)		Decreased (no raw data)	
Goharinia et al.^35^	Sprague-Dawley rats		Decreased (no raw data)		Decreased (no raw data)	
He et al.^37^	Sprague-Dawley rats		Decreased (70–80%)		Decreased (50%–60%)	
Hernández et al.^26^	Sprague-Dawley rats		Decreased (no raw data)			
Liao et al.^32^	C57/BL6 mice	Decreased (no raw data)	Decreased (no raw data)			
Lv et al.^38^	BALB/c Mice	Decreased (50%)				
Nagai et al.^24^	Wistar rats	Decreased (no raw data)	Decreased (no raw data)		Decreased (no raw data)	
Piano et al.^39^	C57BL/6J mice	Decreased (no raw data)	Decreased (no raw data)		Decreased (no raw data)	
Qiu et al.^43^	Brown Norway rats	Decreased (no raw data)	Decreased (no raw data)			
Ren et al.^40^	BALB/c mice	Decreased (no raw data)	Decreased (no raw data)			
Sergeys et al.^31^	C57BL/6J mice	Decreased (no raw data)	Decreased (no raw data)		Decreased (no raw data)	
Tarchick et al.^33^	C57BL/6 mice	Decreased (no raw data)		Decreased (no raw data)	Decreased (no raw data)	
Wang et al.^41^	C57BL/6 mice		Decreased (no raw data)			
Wang and Lo^4^	Sprague-Dawley rats					Decreased (no raw data)
Zhang et al.^34^	Sprague-Dawley rats	Decreased (no raw data)	Decreased (no raw data)		Decreased (no raw data)	

OPs, oscillatory potentials.

#### Total retinal thickness and thickness of individual retinal layers

The majority of selected studies reported retinal thinning after induction of DR^29–31,37–40,43,44^ (Table 4). For example, a decrease of total retinal thickness was assessed either using *in vivo* imaging (OCT) or histology (H&E staining).^31,34,37–40^ However, in 2 studies,^25,43^ an increase in total retinal thickness was found, which authors hypothesized to be caused by retinal edema. In some studies, a decrease of thickness was found in the outer nuclear layer (ONL),^32,34,38–40^ outer plexiform layer,^32,37,39^ inner nuclear layer (INL),^29,32,34,38,40^ inner plexiform layer (IPL),^29,32,37,39^ and ganglion cell layer (GCL)^31,37,38,40^ (Table 4). Zhang et al.^34^ were the only group that reported no changes in IPL thickness in STZ-induced rats, although ONL and INL showed a marked decrease.

**Table 4.** Outcomes of Studies: Total Retinal Thickness and Thickness of Individual Retinal Layers

Study	Species	ONL	OPL	INL	IPL	GCL	Total retinal thickness
Deguchi et al.^25^	Wistar rats						Increased (Healthy control rat 71.0 ± 3.57 μm and STZ-induced 130.6 ± 5.46 μm)
Feng et al.^29^	Sprague-Dawley rats			Decreased (no raw data)	Decreased (no raw data)		
He et al.^37^	Sprague-Dawley rats		Decreased (no raw data)		Decreased (no raw data)	Decreased (no raw data)	Decreased (no raw data)
Liao et al.^32^	C57/BL6 mice	Decreased (no raw data)	Decreased (no raw data)	Decreased (no raw data)	Decreased (no raw data)		
Lv et al.^38^	BALB/c Mice	Decreased (no raw data)		Decreased (no raw data)		Decreased (no raw data)	Decreased (no raw data)
Piano et al.^39^	C57BL/6J mice	Decreased (no raw data)	Decreased (no raw data)		Decreased (no raw data)		Decreased (no raw data)
Qiu et al.^43^	Brown Norway rats						Increased (no raw data)
Ren et al.^40^	BALB/c mice	Decreased (10%)		Decreased (10%)		Decreased (10%)	Decreased (10%)
Sergeys et al.^31^	C57BL/6J mice					Decreased (4%)	Decreased (4%)
Tarchick et al.^33^	C57BL/6 mice						
Zhang et al.^34^	Sprague-Dawley rats	Decreased (no raw data)		Decreased (no raw data)	No changes (no raw data)		Decreased (no raw data)

The raw data were presented by Deguchi et al.^25^ and the decrease in retinal thickness expressed in percentages was shown in other 2 studies.^39,40^ The rest of studies did not provide any raw data (Table 4). Four studies reported a decrease in retinal ganglion cell (RGC) number.^29,31,35,42^ In 2 studies, Sergeys et al.^31^ and Goharinia et al.,^35^ the decrease in RGC number was expressed in percentages (20% and 27%, respectively), whereas other studies^29,42^ did not provide any raw data. In addition, a decrease of cholinergic amacrine cells by 44%,^31^ a lower number of cells in ONL^34^ or lower rhodopsin expression^39^ were separately reported in diabetic animals.

#### Retinal vascular changes

Most of selected studies used either FA, Evans Blue extravasation, extravasation of albumin, or trypsin-digested retinal whole mounts to detect vascular changes in DR-induced rodents^28,29,31,39,41–44^ (Table 5). An increase in vascular permeability was found in the majority of studies.^28,29,41,43,44^ A presence of neovascularization in STZ-injected animals was found in one rat^43^ and one mouse^44^ study, whereas Piano et al.^39^ did not find any neovascular changes in C57BL/6J mice (Table 5). An increase in vascular endothelial growth factor (VEGF) levels were detected in STZ-injected animals of 5 studies.^24,32,37,41,43^

**Table 5.** Outcomes of Studies: Retinal Vascular Changes

Study	Species	Vascular permeability	Neovascularization	Preretinal neovascular cells	VEGF	Acellular capillaries	Vascular endothelial cells	Ghost pericyte
Chang et al.^28^	C57BL/6J mice	Increased (no raw data)				Increased (no raw data)		
Feng et al.^29^	Sprague-Dawley rats	Increased (no raw data)						
He et al.^37^	Sprague-Dawley rats				Increased (no raw data)			
Liao et al.^32^	C57/BL6 mice	Increased (no raw data)		Increased (no raw data)	Increased (no raw data)	Increased (no raw data)		
Nagai et al.^24^	Wistar rats				Increased (9.26-fold higher than in controls)			
Piano et al.^39^	C57BL/6J mice		No changes (no raw data)					
Qiu et al.^43^	Brown Norway rats	Increased (no raw data)	Increased (no raw data)		Increased (no raw data)			
Sergeys et al.^31^	C57BL/6J mice	No changes (no raw data)						
Wang et al.^41^	C57BL/6 mice	Increased (no raw data)			Increased (no raw data)		Increased (no raw data)	
Wang et al.^42^	Sprague-Dawley rats							Increased (no raw data)

Others reported retinal vascular changes, such as an increased formation of acellular capillaries,^28,32^ increased number of vascular endothelial cells,^41^ and an increase of ghost pericytes in diabetic retina.^41^ However, only Nagai et al.^24^ presented raw data from described vascular changes (Table 5).

#### Retinal inflammation and neurodegeneration

The majority of selected studies reported an increase of retinal inflammation and neurodegeneration markers in STZ-injected rodents^26,29,31,37,39,41–44^ (Table 6). For example, 3 studies showed an increase of adherent leukocytes in diabetic retina.^31,41,43^ Sergeys et al.^31^ specified a 2.5-fold increase of adherent leukocytes, 3.1-fold increase of macrophages, and 2.7-fold increase of reactive gliosis as assessed by immunohistochemistry at 8 weeks after STZ injections in C57BL/6J mice. Similarly, a significant increase in the levels of proinflammatory cytokines [tumor necrosis factor-a (TNF-a), interleukin (IL)-1b, IL-6, IL-18, and IL-12] was observed in one study that used C57BL/6J mice.^43^

**Table 6.** Outcomes of Studies: Retinal Inflammation and Neurodegeneration

Study	Adherent leukocytes	Macrophages	Müller cell gliosis	Pro-inflammatory cytokines	VCAM-1 expression	ICAM-1 expression	GFAP expression	BDNF expression
Feng et al.^29^								Decreased to 0.52 ± 0.1-fold
He et al.^37^					Increased (no raw data)		Increased (no raw data)	
Hernández et al.^26^							Increased (GFAP score 1–3)	
Liao et al.^32^				Increased (TNF-a, IL-1b, IL-6, IL-18, IL-12) (no raw data)				
Piano et al.^39^			No changes (no raw data)					
Qiu et al.^43^	Increased (no raw data)					Increased (no raw data)		
Sergeys et al.^31^	Increased (2.5-fold)	Increased (3.1-fold)	Increased (2.7-fold)					
Wang et al.^41^	Increased (no raw data)					Increased (no raw data)	Increased (no raw data)	
Wang et al.^42^							Increased (no raw data)	

At mRNA or protein level an increased expression of glial fibrillary acidic protein (GFAP), which is a marker for reactive gliosis in retina, was observed in 4 studies^26,37,41,42^ and increased expression of vascular cell adhesion molecule-1 (VCAM-1) and intercellular adhesion molecule-1 (ICAM-1) was found in diabetic animals of other 3 studies.^37,41,43^ No changes in Muller glia of diabetic C57BL/6J mice as studied by GFAP immunohistochemistry from retinal sections were reported by Piano et al.^39^ A decreased expression of BDNF as assessed by Western blotting was found in STZ-diabetic Sprague-Dawley rats.^29^

#### Risk of bias

**FIG. 3.**
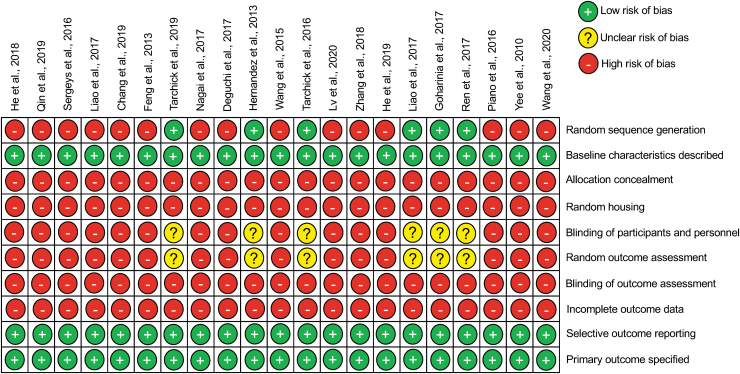
Overview of bias assessment in included studies using the SYRCLE risk of bias tool for animal studies.

All articles specified both the primary outcomes and the baseline characteristics of the study, with sufficient methodological details (Fig. 3). However, only 5% of studies detailed the correct timing of randomization and 24% of studies reported random sequence generation of test subjects. In addition, only 5% of studies reported random outcome assessment and blinding of experiments, but the methodologies were unknown or insufficient to establish suitability. It should be noted that descriptions of methodology did not provide other important parameters of bias, such as sample size calculations, random housing of animals, allocation concealment, or whether gathered data were incomplete and how the researchers dealt with that during the study (Fig. 3).

## Discussion

In this study, we systematically reviewed the literature and included 21 articles that met our inclusion criteria. In all studies, STZ injections were used to induce a phenotype that is reminiscent of DR in rodents. Although the majority of analyzed studies used a single STZ injection, the dose of STZ varied from 30 to 100 mg/kg in rats and from 50 to 150 mg/kg in mice. Furthermore, the follow-up times and study periods varied from 2 to 24 weeks. Glucose level thresholds used to define hyperglycemia were not consistent between studies. However, most studies used either 250 or 300.6 mg/dL blood glucose level as defining criterion. The majority of studies reported a decrease in body weight, whereas only 2 studies^28,41^ found an increase of body weight in diabetic mice; however, the reported body weight gains were lower compared with control groups.

Functional decline of the inner retina is a hallmark of early DR both in humans and in animal models mimicking DR.^45,46^ These early functional deficits can be detected using noninvasive ERG measurement. In this systematic analysis, we found that all analyzed studies reported a deterioration of visual function as assessed either locally using ERG or at the level of occipital cortex using VEP. However, only 2 studies reported a percentage change in which a-wave, b-wave, and OP amplitudes decreased after DR induction compared with healthy control animals.^37,38^ Other studies did not provide raw data. Therefore, we could not compare and conclude on the magnitude of visual function decrease in our analysis.

Reduced total retinal thickness or reduced thickness of individual retinal layers in DR suggests neuronal degeneration. Our systematic review revealed that the majority of included studies reported a decrease in total retinal thickness. Interestingly, total retinal thickness was increased in 2 studies on Wistar^25^ and Brown Norway rats,^43^ which most likely indicates retinal edema. Similarly, as with functional data, only 2 studies provided raw data on the total retinal thickness or individual retinal layers, which were expressed as percentage.

DR is characterized as a vascular disease that is manifested by endothelial cell proliferation, increased vascular permeability,^47,48^ and inflammatory response.^49^ In this systematic review we found that the majority of analyzed studies used either FA, Evans Blue extravasation, extravasation of albumin, or trypsin-digested retinal whole mounts to detect vascular changes in DR-induced rodents.

Selected studies detected various vascular changes in STZ rodents, including increased vascular permeability, neovascularization, increase in VEGF levels, appearance of acellular capillaries, vascular endothelial cells, and ghost pericytes. These vascular changes were accompanied with inflammatory events: increased number of leukocytes, increased reactive gliosis of Muller cells, release of inflammatory cytokines and increased number of macrophages, and increased expression of endothelial cell adhesion molecules, such as ICAM-1 and VCAM-1.

Our detailed risk of bias analysis revealed that only less than one-third of studies (29%) reported the use of randomization and random sequence generation to assign the interventions. Furthermore, methods of blinding were inadequate or not reported at all. To confound this further, random housing of animals or treatment allocation concealment was also missing from all studies. Whether this is due to inadequate reporting or a lack of use of these methods when performing animal experiments is not clear. However, a failure to carry out these methods when conducting animal experiments is concerning, since randomization is essential for obtaining comparable study groups and may confound study findings.^50,51^

The inadequate number of studies reporting correct timing of randomization is also worrying since the lack of randomization together with the lack of allocation concealment, particularly in unblinded studies, could lead to variations in the preintervention study conditions and potentially affect the outcomes.

The lack of blinding in the majority of studies (71%) is a fundamental shortcoming and raises doubts about data validity.^50,52^ Random outcome assessment was again missing in the majority of the studies (71%) and the methodology was unknown or insufficient. In addition, attrition bias was difficult to assess in these studies since none of the studies specifically reported on this aspect of the risk of bias tool. Hence, we left this as not reported to highlight the issue and the potential bias that this could introduce. Finally, none of the studies reported sample size calculations, which is another confounder introducing bias into these studies. For example, too few animals may not be sufficient to detect treatment effects (e.g., binary outcomes such as survival, etc.), whereas the use of too few and too many animals are regarded as an unethical waste of resources.^53,54^

Especially given the utility of the STZ model for drug discovery for DR, we urge researchers to adhere to a minimum reporting standard, for example by adhering to the “Animal Research: Reporting of *In Vivo* Experiments” (ARRIVE) and ARRIVE 2.0 guidelines and reporting the “ARRIVE Essential 10,” which are the recommended minimum requirement for reporting animal experiments.^55,56^ The “ARRIVE Essential 10” include a detailed description of study design, sample size, inclusion and exclusion criteria, blinding, outcome measures, statistics, identification of experimental animals, description of experimental procedures, as well as recommendations for how to report and present the results^.55,56^ Consideration of these recommendations in the reporting of STZ studies would mitigate many of the reporting shortcomings of the studies reviewed herein.

### Limitations

The leading limitation of this study was that due to the lack of quantitative data in the included studies, meta-analysis was not possible to perform. The lack of quantitative data for all of the outcome measures in the majority of the studies significantly hampers the interpretation of obtained results. Simply reporting that the values “decreased” or “increased” in diabetic animals does not provide helpful insight on the magnitude of changes. Quantitative data would have been easier to compare and would provide more information on determining the efficacy of a particular outcome in DR. A possible limitation could also arise from differences in follow-up times ranging from 2 to 24 weeks, and a wide range (150–350 mg/dL) of blood glucose levels, which were used to classify animals into diabetic and nondiabetic.

## Conclusions

This systematic review demonstrates that the most consistent reported readout in the STZ model is the functional assessment of visual function by ERG. Specifically, amplitudes of a-wave, b-wave, and OP correlated with DR-related pathology. However, a significant concern is the relatively high risk of bias in the reviewed studies, either due to the lack of reporting or lack of correctly following procedures to reduce bias, thereby limiting the potential usefulness of these studies. Stricter adherence to the ARRIVE guidelines are recommended in future studies. Our review, therefore, highlights a need for further studies, considering the risk of bias, to validate that ERG is the most useful readout in preclinical studies of DR.
